# Optimizing use of multi-antibody assays for Lyme disease diagnosis: A bioinformatic approach

**DOI:** 10.1371/journal.pone.0253514

**Published:** 2021-09-09

**Authors:** Richard Porwancher, Lisa Landsberg

**Affiliations:** 1 Division of Infectious Diseases, Department of Medicine, Rutgers Robert Wood Johnson Medical School, New Brunswick, New Jersey, United States of America; 2 Infectious Disease Consultants, PC, Mercerville, New Jersey, United States of America; 3 Clinical Research Operations & Regulatory Affairs, Lewis Katz School of Medicine, Temple University, Philadelphia, Pennsylvania, United States of America; University of Toledo College of Medicine and Life Sciences, UNITED STATES

## Abstract

Multiple different recombinant and peptide antigens are now available for serodiagnosis of Lyme disease (LD), but optimizing test utilization remains challenging. Since 1995 the Centers for Disease Control and Prevention (CDC) has recommended a 2-tiered serologic approach consisting of a first-tier whole-cell enzyme immunoassay (EIA) for polyvalent antibodies to *Borrelia burgdorferi* followed by confirmation of positive or equivocal results by IgG and IgM immunoblots [standard 2-tiered (STT) approach]. Newer modified 2-tiered (MTT) approaches employ a second-tier EIA to detect antibodies to *B*. *burgdorferi* rather than immunoblotting. We applied modern bioinformatic techniques to a large public database of recombinant and peptide antigen-based immunoassays to improve testing strategy. A retrospective CDC collection of 280 LD samples and 559 controls had been tested using the STT approach as well as kinetic-EIAs for VlsE1-IgG, C6-IgG, VlsE1-IgM, and pepC10-IgM antibodies. When used individually, the cutoff for each kinetic-EIA was set to generate 99% specificity. Utilizing logistic-likelihood regression analysis and receiver operating characteristic (ROC) techniques we determined that VlsE1-IgG, C6-IgG, and pepC10-IgM antibodies each contributed significant diagnostic information; a single-tier diagnostic score (DS) was generated for each sample using a weighted linear combination of antibody levels to these 3 antigens. DS performance was then compared to the STT and to MTT models employing different combinations of kinetic-EIAs. After setting the DS cutoff to match STT specificity (99%), the DS was 22.5% more sensitive than the STT for early-acute-phase disease (95% CI: 11.8% to 32.2%), 16.0% more sensitive for early-convalescent-phase disease (95% CI: 7.2% to 24.7%), and equivalent for detection of disseminated infection. The DS was also significantly more sensitive for early-acute-phase LD than MTT models whose specificity met or exceeded 99%. Prospective validation of this single-tier diagnostic score for Lyme disease will require larger studies using a broader range of potential cross-reacting conditions.

## Introduction

Lyme disease (LD) is the most common tick-borne disease in the United States and constitutes an increasing public health threat; the CDC now estimates that 476,000 Americans are diagnosed and treated yearly [1]. Ninety-five percent of cases are concentrated in 14 states in the Northeast, Mid-Atlantic, and upper Midwest regions [2]. Early diagnosis of LD relies on identification of the skin lesion called erythema migrans (EM), while diagnosis of cardiac, neurologic, and rheumatologic manifestations is dependent on both clinical criteria and laboratory methods [2]. Measuring serum antibodies to *Borrelia burgdorferi*, the causative agent, is the most common diagnostic approach for disseminated infection. Since 1995 the CDC has advocated a 2-tiered serologic method consisting of a first-tier EIA or immunofluorescent antibody assay (using either whole-cell or *B*. *burgdorferi*-derived peptide antigens), followed by IgG and IgM Western blot confirmation of positive or equivocal first-tier results [3]. After the first 30 days of illness, only IgG immunoblots are used for confirmation. While at least 90% sensitive for late-stage disease [4], the standard 2-tiered approach (STT) is limited by low sensitivity for early LD (38% to 50%) and false-positives secondary to subjective interpretation of IgM immunoblot bands and cross-reacting antibodies (1% to 8%) [4–7]; these drawbacks have led to a search for more sensitive and specific alternatives.

Between 70% and 90% of all *B*. *burgdorferi* infections lead to EM, typically appearing between 3 and 30 days following an *Ixodes scapularis* tick bite (*Ixodes ricinus* in Europe) [8, 9]. Patients presenting with EM in an endemic setting should generally be treated with antibiotics rather than undergo serologic testing [10]. EM diagnosis, however, is imperfect. Primary care physicians practicing in endemic communities can identify approximately 70% of patients with typical EM, sometimes confusing it with allergic reactions to insect bites as well as other dermatologic conditions [11]. An EM-like skin lesion called “southern tick-associated rash illness” (STARI) may be the consequence of an infectious agent spread by the lone-star tick, *Amblyomma americanum* [12]; although most STARI cases have been diagnosed in the South-Central and Southeastern US, the geographic range of *Ambylomma americanum* overlaps with *Ixodes scapularis* and STARI lesions can be mistaken for EM secondary to *B*. *burgdorferi* [12]. Fortunately, STARI does not appear to generate antibodies that cross-react with *B*. *burgdorferi* [12]. Between 7% and 16% of patients with early LD experience either delayed diagnosis or present with an influenza-like illness without EM [13–15] and up to 9% demonstrate atypical EM lesions [16]. Physician education regarding diagnosis of early disease is both important and useful [11], but may not be sufficient [17]. Between 13% and 27% of patients with early-acute-phase neurological disease may be either seronegative or fail to satisfy current STT guidelines [18–21]. Both physicians and the public are aware that the incidence of Lyme disease has increased in recent years [1, 2]. Because of its expanding geographic range, public fear of contracting LD, and diagnostic uncertainty among inexperienced physicians, there is a significant demand for LD serology [22, 23]. Given the challenges associated with clinical diagnosis of EM and limitations in STT performance, there remains a need for more accurate diagnostic tests, even for early Lyme disease.

Much research over the last 20 years has been devoted to finding *B*. *burgdorferi* antigens that elicit a broad and early immune response; three of the most promising have been C6, VlsE, and pepC10 [24–26]. The most immunogenic antigen is C6, a 26-mer peptide representing the 6^th^ invariant portion of VlsE (variable major protein-like sequence expressed) [25]. The immune response to C6 is both early and largely IgG-mediated [25, 27]. VlsE is a highly immunogenic *B*. *burgdorferi* surface antigen important in immune evasion that generates both IgG and IgM immune responses [27]. OspC is a *B*. *burgdorferi* surface antigen that enables dissemination through attachment to host plasminogen [28]; it generates one of the earliest IgM-based immune responses [26]. A 10-mer peptide from the invariant N-terminal portion of OspC, called pepC10, has been noted to generate an immune response with somewhat better performance characteristics for neurological disease than its parent protein [26]. Commercial assays were developed that detect IgG and IgM antibodies to C6 by EIA (Oxford Immunotec, Boston, MA) and IgG and IgM antibodies to VlsE by chemiluminescent assay (CLA) (Liaison ^®^ assay, DiaSorin, Saluggia, Italy). While excellent for detection of disseminated infection, the latter 2 assays demonstrate approximately 40% false-negatives in early-acute-phase sera from patients with EM [29] and false-positive serology in 2% to 5% of controls [29, 30]; Western blot confirmation of positive or equivocal results is still recommended to improve specificity [30].

Attempts to use broader antibody panels simultaneously have demonstrated improved sensitivity but at a cost of lower specificity [24, 31–34]. Most multi-antibody assays use Boolean “OR’ logic for test interpretation (i.e., the overall test is considered positive if any individual component antibody exceeds its cutoff). Because each antibody may demonstrate its own set of false-positive responses, the total number of sera demonstrating false-positive reactions typically increases as the number of assays increase [31–34]. If Boolean “AND” logic is used for test interpretation, then detecting 2 or more antibodies in the same sample is required to consider a test positive; the latter approach improves specificity, but may reduce sensitivity [35]. Lahey et al. [36] proposed a broad multi-antibody panel of highly specific short peptide antigens; despite requiring 2 or more antibodies for positive results, the broad panel permitted good sensitivity. The latter approach requires a much larger control population and prospective confirmation before widespread adoption.

More recently a modification of the standard 2-tiered serologic approach has been proposed that employs a second-tier EIA test rather than immunoblotting to confirm positive or equivocal first-tier EIA results [5, 37, 38]. Either a whole-cell EIA or the Liaison assay for VlsE-IgG/IgM antibodies has been used as the first-tier of an all-EIA approach, followed by the Oxford Immunotec C6-IgG/IgM EIA to confirm first-tier results [5, 29, 37, 39, 40]. Because antibodies to the C6 peptide often appear before Western blotting becomes positive, these modified 2-tiered (MTT) approaches have demonstrated better sensitivity than the STT for early LD with equivalent specificity [21, 24, 37]. Due to automation, MTT approaches are also more cost-effective and objective than immunoblotting [38, 41]. Recently a MTT all-EIA assay manufactured by Zeus Scientific (Branchburg, NJ) was cleared by the FDA as an alternative to the STT, demonstrating equivalent overall performance and possibly better sensitivity for early LD [42, 43]. The first tier of the Zeus assay measures IgG and IgM antibodies to recombinant VlsE1 and pepC10 by standard EIA; positive or equivocal first-tier results are confirmed using a whole-cell EIA that detects either polyclonal antibodies or separate IgG and IgM antibodies to *B*. *burgdorferi*. According to Bayes’ Theorem the sequence of the Zeus MTT can be reversed without change in performance, provided that positive and equivocal results are treated the same. A single unpublished retrospective cohort study compared the Zeus MTT to the STT in 30 patients with acute erythema migrans; MTT sensitivity using a second-tier EIA for polyclonal antibodies was 73.3% in early-acute-phase sera versus 50% using the STT [43]. The latter results need prospective confirmation in clinically well-defined populations, but may not be sufficient to exclude early-acute-phase disease.

While the above MTT approaches have been welcome alternatives to the STT [5], they too utilize Boolean “AND” logic since both EIA tests must be positive or equivocal to consider the overall test positive; MTT test sensitivity may therefore be lower than that achievable using “OR” logic [29, 38]. Modern bioinformatics may provide guidance to help overcome the above limitations with multi-antibody and multi-tiered approaches. Valuable information may be lost when assays that provide numerical results (i.e., continuous variables) are reported as categorical results (i.e., either positive or negative); multiple different LD immunoassays have demonstrated that the higher the antibody level, the more predictive the assay [30, 44, 45]. Also, some antibodies may be more important diagnostically than others [45]. Bioinformatic algorithms have been used to interpret multi-antibody assay results by assigning a diagnostic value (weight) to each antibody result [34, 35, 45–48]. For a given antibody in a given patient sample, its diagnostic contribution is the product of its serum level multiplied by its diagnostic weight. A diagnostic weight can also be assigned to a categorical result (e.g., presence or absence of an immunoblot band). The weighted contributions of each assay are typically added together in a linear fashion, creating an overall diagnostic score (DS) [49]. Logistic regression analysis is the most common biostatistical technique used to assign weights to important variables in a linear model diagnostic score [50]; diagnostic scores can also be generated using non-linear models [50, 51]. A cutoff is chosen for the overall score, not the individual assays, to achieve a clinically acceptable false-positive rate [48]. Provided that at least one numerical assay is included in a diagnostic panel and provided that the likelihood of disease increases as the numerical value of each such assay increases, the Neyman-Pearson Lemma, a mathematical proof, states that a weighted diagnostic score optimizes the likelihood of disease for any given false-positive rate [49, 50]; for multi-antibody panels that meet the above conditions, this means that diagnostic scores can achieve sensitivity that is greater than or equal to all other interpretive methods, including multi-tiered approaches. Bioinformatic algorithms have the potential to utilize the information provided by multi-antibody assays more effectively than standard interpretive methods.

Receiver operating characteristic (ROC) curves plot the trade-off between a test’s sensitivity and specificity as its cutoff is varied; in general, the greater the area under the ROC curve (AUC), the better the assay. Diagnostic scores that maximize the AUC within a given range of specificity also maximize test sensitivity within that range [50], but there exist challenges to achieving this goal. Since test specificity may be clinically relevant only within a limited range (e.g., ≥ 80% specificity), statistical techniques that maximize only a portion of the ROC curve may be preferred [48, 51]. When designing a multi-antibody approach, investigators must now choose from among a broad panel of potential antibodies for LD diagnosis [35, 52, 53]. One approach is to utilize only the most sensitive antibodies to generate a diagnostic score [53, 54]; panels developed using the latter approach may not recognize antibodies that are diagnostically complementary, even if some assays are less sensitive than others [48]. Also, panels that utilize separate diagnostic scores for IgG and IgM antibodies [35, 55, 56] might miss potentially complementary isotype responses to diagnose a given disease stage [45, 48]. However, including all potential antibodies in a single panel may be equally problematic because some assays are diagnostically redundant, potentially reducing overall specificity [45] and raising costs; careful step-wise elimination of assays from antibody panels while rechecking the AUC at each step is usually necessary to optimize diagnostic score performance [57]. In addition to individual variables, statistical interaction terms (represented by the multiplication product of two variables) can be included in a diagnostic score; these terms essentially act like another variable [58]. While existing literature suggests that diagnostic scores may help improve sensitivity for early LD when compared to the STT [34, 48, 54], even better score performance may be possible by incorporating the statistical techniques described above.

Although there is a strong theoretical basis for using bioinformatic diagnostic scores, they have not yet been empirically compared to modified 2-tiered (all-EIA) approaches using the same panel of recombinant and peptide-antigens. Head-to-head comparisons of STT and MTT approaches against a single-tier diagnostic score are therefore relevant to optimizing LD serodiagnosis. A prior study by Bacon et al. [24] compared the performance of the STT to a panel of IgG and IgM kinetic-EIA antibody assays that utilized VlsE1, C6 peptide, and pepC10 antigens; this study represents the largest public database of Lyme disease serology in the US [24]. These authors determined that the 2-antibody combination of C6-IgG plus pepC10-IgM was significantly more sensitive than the STT, but also less specific. Applying modern bioinformatic techniques, we re-evaluated this same database to explore multi-antibody diagnostic scores and modified 2-tiered approaches not previously reported; our results demonstrated that diagnostic scores performed significantly better than STT and MTT approaches for diagnosis of early LD.

## Methods

### Patient population

The Bacon data set [24] consists of test results from 839 retrospectively-collected serum and plasma samples, including 280 samples from LD patients and 559 controls. Because 97% of samples were serum specimens, we hereafter refer to these samples collectively as “sera.” Study sera had been collected prior to 2003 and all LD patients met the 1990 CDC case surveillance definition [59]. There were 80 early-acute-phase sera from patients with EM, 106 early-convalescent-phase sera from EM patients, and 94 sera from patients with extracutaneous disease, including arthritis (33 acute and 24 convalescent sera), and neurological disease (15 early-acute, 11 early-convalescent, and 11 late-stage sera). Patients with multiple LD manifestations were assigned to only one group based on their physician’s principal diagnosis. Most EM lesions were culture-positive for *B*. *burgdorferi* by skin biopsy. Sample sources are described below and sera were stored at -20 degrees C prior to testing.

#### Erythema migrans (early Lyme disease)

Tufts New England Medical Center contributed 77 samples from patients with EM, including 37 pairs of early-acute- and early-convalescent-phase sera; the interval between disease onset and initiation of medical treatment ranged from 1 to 8 weeks. The median interval between early-acute- and early-convalescent-phase sera was 18 days (range: 8 to 43 days). The 3 unpaired sera from Tufts consisted of 1 early-acute- and 2 early-convalescent-phase samples. New York Medical College provided 82 samples from patients with culture-confirmed EM, consisting of 41 pairs of early-acute- and early-convalescent-phase sera; the median interval between disease onset and acute-phase sera was 3 days (range: 0 to 67 days) and the median interval between early-acute- and early-convalescent-phase sera was 11 days (range: 7 to 106 days). The CDC Division of Vector-Borne Diseases contributed one early-acute- and 26 early-convalescent-phase samples from patients with EM, including two with heart block; excluding one CDC patient with missing data, the median duration between the start of antibiotic treatment and collection of early-convalescent-phase sera was 55 days (range: 16 to 227 days). A total of 55% of all EM-related sera were from patients whose skin lesions were culture-positive. Forty-six percent of all early-acute-phase sera were collected within 1 week of disease onset and 85% within 4 weeks.

Forty-eight percent of early-acute-phase sera and 43% of early-convalescent-phase sera were from patients with multiple EM lesions. The serologic database from the Bacon study [24] is available as (S1 Data) but did not list the serology from patients with single and multiple EM lesions separately. Even though patients with multiple EM are thought to have a form of disseminated infection [9], we were unable to analyze their serologic response separately from those with single EM lesions. For purpose of our analyses, early LD refers to all patients with EM and disseminated infection refers to those with extra-cutaneous disease manifestations.

Although now thought to represent 7% to 16% of all early LD cases [8, 13, 14] our study population did not include patients with an “influenza-like” presentation of LD; these patients typically develop a febrile illness in the late spring or early summer without EM or significant respiratory or GI complaints. Patients were not systematically evaluated for tick-borne co-infections.

#### Early neurological disease

A total of 20 samples were provided by Tufts New England Medical Center, consisting of 15 acute-phase and 5 convalescent-phase sera from patients with early neurological disease. Eighteen patients (90%) had physician-diagnosed erythema migrans, 14 had meningitis, 9 had facial palsy, and 5 had radiculoneuropathy (patients could demonstrate more than 1 disease manifestation simultaneously). The CDC contributed an additional 6 convalescent-phase samples, including 5 with facial palsy and 1 with meningitis and radiculopathy; three of the above patients had physician-diagnosed EM and one had heart block.

#### Late neurological disease

Nine samples provided by Tufts came from patients with varied neurological manifestations, including 7 with Lyme encephalopathy and 3 with polyneuropathy; 6 had a history of EM. Seven Tufts patients had received antibiotic therapy before specimen collection, including 5 treated for Lyme arthritis before developing neurological disease. Two samples from patients with Lyme encephalopathy were contributed by the National Institute of Allergy and Infectious Diseases (NIAID) of the National Institutes of Health.

#### Lyme arthritis

Tufts New England Medical Center contributed 48 sera from patients with Lyme arthritis, including 27 pre-treatment and 21 convalescent-phase sera; 5 of the latter cases had failed antibiotic therapy. Thirty patients had a history of EM. The CDC contributed 8 convalescent-phase samples and the NIAID contributed 1 pre-treatment sample.

#### Controls

The CDC provided 559 control samples, including 257 from healthy individuals (243 from non-endemic and 14 from endemic communities). A total of 302 sera represented potentially cross-reacting conditions, including anti-cardiolipin antibodies (15), anti-nuclear antibodies (116), leptospirosis (10), multiple sclerosis (10), asymptomatic rapid plasma reagin-positive sera (14), primary and secondary syphilis (4), latent syphilis (10), asymptomatic rheumatoid factor-positive sera (15), rheumatoid arthritis (94), and tick-borne relapsing fever (14).

### Kinetic-EIA and antigen production

A kinetic-EIA was performed by Bacon et al. [24] to measure IgG and IgM antibodies to recombinant VlsE1, IgG antibody to the C6 peptide, and IgM antibody to pepC10; the kinetic-EIA technique employed was described therein and we refer the reader to that text. The kinetic-EIA reaction rate was calculated by measuring the optical density of the *p*-nitrophenol phosphate substrate at 405 nm every 2 minutes over a 10-minute period. The dynamic range of individual kinetic-EIA assays was between 2 and 3 logs. Binary test cutoffs for VlsE-IgG, C6-IgG and pepC10-IgM antibodies were selected to yield a 1% false-positive rate for each antibody among the 559 controls. A brief description of the antigens used is detailed below.

### VlsE1 production

Recombinant VlsE1 antigen (variable major protein-like sequence 1) was produced at the CDC, Fort Collins, CO. The *vlsE1* allele from *Borrelia burgdorferi* B31 was amplified by PCR and transformed into protease-deficient *Escherichia coli* strain BL21 using a pTYB2 plasmid vector. The gene fusion protein included a chitin-binding intein tag. After protein synthesis and cell lysis, the protein product was isolated by single-column affinity chromatography using the intein tag and then eluted using a dithiothreitol cleavage buffer. Recombinant VlsE1 purity was demonstrated by SDS-PAGE stained with Coomasie blue, indicating no residual N-terminus vector-derived residues.

### C6 production

A 26 amino acid sequence from the IR6 region of the IP90 strain of *Borrelia garinii*, CMKKDDQIAAAMVLRGMAKDGQFALK, was synthesized by Louisiana State University Medical Center core laboratory (New Orleans) using Fmoc chemistry and biotinylated at the amino terminus using N-hydroxysuccinimide biotin. The peptide sequence was verified by MALDI-TOF and amino acid analysis.

### pepC10 production

The pepC10 amino acid sequence PVVAESPKKP was synthesized using Fmoc chemistry by the CDC Biotechnology Core Facility at Atlanta, GA. A biotinylated 6-carbon spacer was introduced at the amino end of pepC10 using sulfo-NHS-LC-biotin and the protein’s identity was confirmed by MALDI-TOF.

### Standard two-tiered serologic testing

A whole-cell EIA for both IgG and IgM antibodies was performed for each sample using the VIDAS^®^ Lyme IgG and IgM (LYT) EIA (bioMerieux Vitek system, Durham, NC); test results were reported on a continuous scale and interpreted according to the manufacturer’s guidelines. Positive or equivocal VIDAS EIA results were confirmed by IgG and IgM Western blotting (MarDx, Carlsbad,CA). Manufacturer’s guidelines were used for Western blotting and current CDC-advocated standards were used for interpretation [3]. A positive IgM Western blot required the presence of at least two of the three following bands: 23-, 39-, or 41-kDa. A positive IgG Western blot required the presence of at least 5 of the following 10 bands: 18-, 23-, 28-, 30-, 39-, 41-, 45-, 58-, 66-, or 93-kDa. For EIA-positive or–equivocal patient sera obtained less than 30 days after disease onset, either IgG or IgM immunoblot confirmation was sufficient for diagnosis; except for 2 patients, sera obtained more than 30 days after disease onset required a positive IgG immunoblot for diagnosis. The latter two patients, one with facial palsy and EM and the other with Lyme arthritis and EM, each had only one serum sample collected >30 days after starting antibiotic treatment; they were considered 2-tier positive using IgM blot criteria alone by the original investigators [24]. Control samples with either EIA-positive or–equivocal test results could be confirmed by either IgM or IgG immunoblotting.

### Statistical methods

#### Binary antibody models, diagnostic scores, and ROC curves

Analyses were performed using MATLAB statistical software, version 7.2.0.232 (R2006a) (MathWorks, Boston, MA) except as specified below. The cutoff for the VIDAS EIA was chosen by its manufacturer. Cutoffs for each kinetic-EIA were chosen to generate 99% specificity among the overall control population. Individual antibody assays were interpreted as either positive or negative based on a single cutoff and were called “binary assays.” Models that utilized multiple binary assays were called “binary models.”

The construction of binary models is empiric and we selectively used Boolean “AND” and “OR” logical operators to maximize sensitivity while preserving specificity. One of the principal goals of our research was to employ multiple kinetic-EIA antibody assays to expand test sensitivity, a goal facilitated by using Boolean “OR” logic for test interpretation. Binary models employing Boolean “OR” logic were, therefore, constructed using all possible combinations of 2 or more kinetic-EIAs; these binary models were evaluated for use either as a single-tier approach or as the second tier of a 2-tiered approach. Two-tiered models perform tests in sequence, requiring the first test to be either positive or equivocal before performing the second test; this approach is mathematically equivalent to using Boolean “AND” logic for interpretation. Because a 2-tiered model uses “AND” logic for interpretation, it can only be as sensitive as its least sensitive tier. First-tier (screening) assays are typically chosen to achieve high sensitivity while confirmation by second-tier assays is needed to maintain high specificity. The VIDAS EIA was the most sensitive individual assay in our study population and was preferentially used as the first-tier of a 2-tiered approach. Similar to Lahey at al. [36], we explored applying a combination of logical operators to the entire set of EIAs (VIDAS EIA and kinetic-EIAs); the latter single-tier approach required 2 or more positive/equivocal assays among this group to consider the overall test positive.

To generate diagnostic scores, assays that produced numerical results were first normalized by dividing individual antibody results by their standard deviation. Utilizing the VIDAS EIA as well as VlsE1-IgG, C6-IgG, VlsE1-IgM, and pepC10-IgM kinetic-EIA antibody levels, ordinal logistic regression models were derived using step-wise variable selection with backward elimination (two-tailed alpha = 0.05); maximum likelihood estimates were generated using the Levenberg modification of the Newton method with the object of maximizing the area under the ROC curve (AUC). One statistical interaction term was added to enhance model performance when 3 or more antibodies were included in the panel; all possible pairs of antibody results were empirically modeled to identify the interaction term(s) that maximized the AUC. Regression models generated a diagnostic score for each sample, consisting of a weighted linear combination of individual antibody results with the general form:

$$Diagnostic\;score={(\beta }_{1})∙{(Antibody\;level}_{1})+{(\beta }_{2})∙{(Antibody\;level}_{2})+\cdots +{(\beta }_{n})∙{(Antibody\;level}_{n})+{(\beta }_{j,k})∙{(Antibody\;level}_{j})∙({Antibody\;level}_{k}),$$

where (*β*_*i*_) represents the beta-coefficient (weight) assigned to the *i*^*th*^ antibody level, *i* ∈ (1, 2, …, *n*), where *j* and *k* represent the antibodies used for the interaction term, *j*, *k* ∈ (1, 2, …, *n*), *j* ≠ k, and where *β*_*j*,*k*_ represents the beta-coefficient (weight) of the interaction term.

A ROC curve was generated for each model by altering the DS cutoff value; the AUCs of different models were compared using a cross-validated, 2-fold holdout bootstrap technique with 500 replications to generate 95% confidence intervals (CI) [60]; each bootstrap iteration involved training on one sample and testing on the hold-out sample. When a binary model used only two antibodies, a diagnostic score using these same two antibodies was generated using the above logistic regression technique. The sensitivity and specificity of each binary model were functions of the performance characteristics of its individual components and were reported as proportions. The 95% confidence interval for differences in proportions for paired data was calculated using the Wilson method without continuity correction [61]; Wilson confidence intervals were calculated using R statistical software, version 3.6.2 [62]. To be considered statistically significant, the 95% confidence interval of the difference between AUCs or proportions could not include zero (two-tailed alpha = 0.05).

While there was some latitude in choosing a cutoff for the diagnostic score, it was always set to generate specificity greater than or equal to the STT (99%). Because of extensive overuse of diagnostic tests for Lyme disease, public health considerations require that viable alternatives to the STT demonstrate equivalent specificity [63]. For each diagnostic score cutoff the resulting sensitivity and specificity were rounded to the nearest whole numbers to generate proportions. To compare model sensitivities the diagnostic score cutoff was chosen to approximate a binary model’s specificity and then sensitivities were compared using the Wilson method. Alternatively, model specificities could be compared after setting the diagnostic score cutoff to approximate binary model sensitivity. We chose not to produce ROC curves for the STT or binary models because they would have been represented by only a single point. The primary study goal was to identify the multi-antibody model that generated the greatest improvement in sensitivity compared to the STT without loss of specificity (≥99%). Secondary goals included comparing diagnostic scores to modified 2-tiered approaches and comparing model performance by disease stage. Analyses of early LD, whether early-acute-phase or early-convalescent-phase, used only sera from patients with EM; analyses of disseminated infection, whether acute-phase or convalescent-phase, used only sera from patients with neurological disease or Lyme arthritis.

#### Disease stage-specific models

A limited comparison of disease stage-specific models was performed for early-acute-phase disease, early-convalescent-phase disease, and disseminated disease. Utilizing the same statistical techniques, the diagnostic weights assigned to VlsE1-IgG, C6-IgG, and pepC10-IgM antibodies and interaction term(s) were chosen to optimize performance for each disease stage. The cutoffs for the diagnostic scores optimized for each disease stage were chosen to generate 99% specificity. The sensitivity of each stage-specific model was compared to the STT as well as the sensitivity of the diagnostic score standardized using the entire dataset; the Wilson method was used to compare proportions.

#### Single-tier versus modified two-tiered approaches to Lyme disease diagnosis

**Fig 1:**
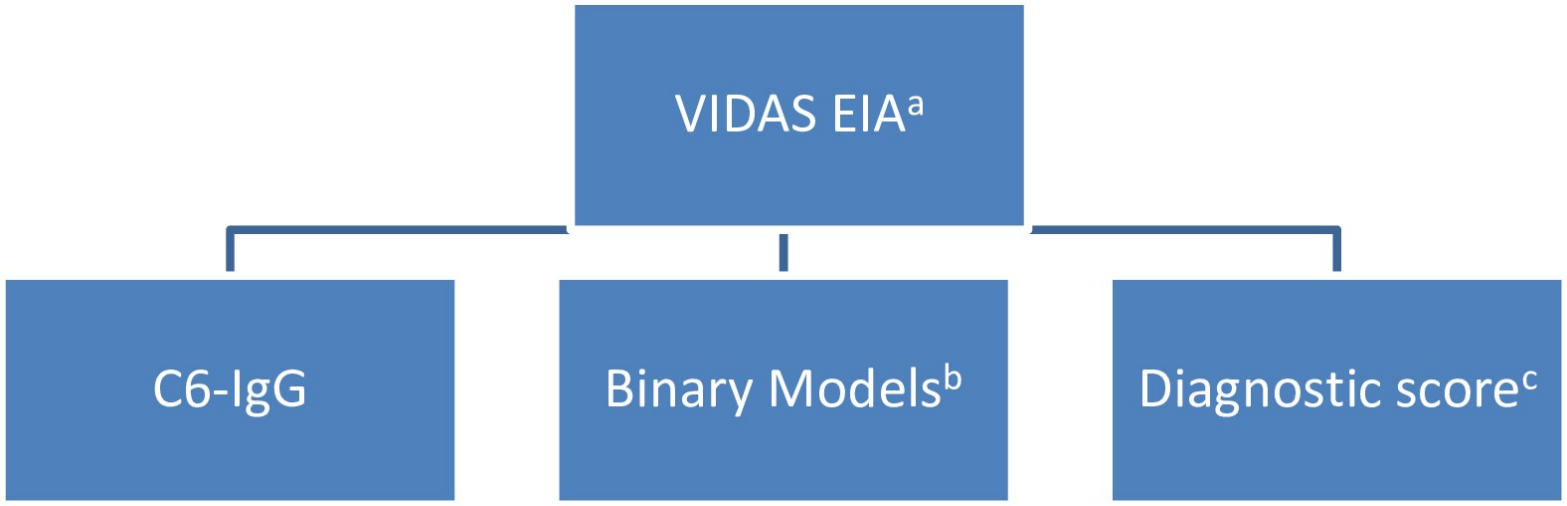
Testing algorithm for modified 2-tiered approaches to Lyme disease diagnosis. ^a^ Positive or equivocal VIDAS EIA results were confirmed by a second-tier EIA method. ^b^ See Table 2 for a complete list of binary antibody models evaluated. ^c^ Diagnostic score optimized as described above.

Because the 2-antibody binary models reported by Bacon et al. [24] demonstrated lower specificity than the STT, we explored alternative models using modified 2-tiered (MTT) approaches and diagnostic scores. MTT approaches utilized a second-tier EIA (or combination of EIAs) to confirm positive or equivocal first-tier EIA results. Diagnostic scores were evaluated both as a single-tier approach and as the second tier of a MTT approach. Utilizing the VIDAS EIA as a first-tier antibody assay, we confirmed positive or equivocal results using each of the following: C6-IgG antibody by kinetic-EIA, select binary models using the kinetic-EIAs described above, and an optimized diagnostic score using the same kinetic-EIAs (Fig 1). When a binary model was used as the second tier of a MTT approach, we called this a MTT binary model. We also explored utilizing the C6-IgG antibody kinetic-EIA as the first-tier of a MTT approach; positive or equivocal C6-IgG antibody EIA results were confirmed using binary models composed of the remaining kinetic-EIAs. The sensitivities of MTT binary models were compared to each other and to the STT at equivalent specificity (≥99%). We also compared the diagnostic score, used as a single-tier approach, to MTT binary models and the STT. The purpose of these models was to find the best alternative to the STT from among the antibody assays available to us, whether a MTT approach or diagnostic score. Statistical differences in proportions were described using 95% and 99% confidence intervals calculated by the Wilson method for paired data without continuity correction [62].

## Results

Logistic-likelihood regression analysis was performed using the entire dataset; Table 1 details the beta-coefficients and standard errors generated for VlsE1-IgG, C6-IgG, pepC10-IgM, VlsE1-IgM antibodies by kinetic-EIA and antibodies to whole-cell lysate by VIDAS EIA. The impact of step-wise elimination of each assay from the model was assessed by measuring the area under the ROC curve (AUC). Eliminating both the VIDAS EIA and VlsE1-IgM antibody assays from the model did not significantly reduce the AUC, but removing the VlsE1-IgG, C6-IgG, or pepC10-IgM antibody assays led to statistically significant losses (0.7%, 4.6%, and 1.0%, respectively). The optimal assay panel for our dataset therefore consisted of VlsE1-IgG, C6-IgG, and pepC10-IgM antibodies. A diagnostic score was generated for each sample using a weighted linear combination of the individual antibody levels to these 3 antigens and one statistical interaction term (i.e., multiplication product of the levels of two different antibodies); of the 3 possible pairs of antibodies, the diagnostic score that included a (C6-IgG)·(pepC10-IgM) interaction term generated the greatest AUC. The beta-coefficients of the final model were 0.061 for VlsE1-IgG, 0.257 for C6-IgG, 0.030 for pepC10-IgM and 0.007 for the interaction term, yielding an AUC of 0.976 for this model. Although diagnostic score cutoffs can be chosen using decision-analytic techniques [64], the latter approach is beyond the scope of the current study. We instead explored diagnostic score cutoffs that met or exceeded STT specificity (≥ 99%), emphasizing the importance of specificity as a public health goal [48, 63]. At 99.1% specificity, the diagnostic score’s sensitivity was 79.6%.

**Table 1:** Beta-coefficients and standard errors of study antibody panel using logistic-likelihood regression analysis^a^.

Antibody	VlsE1-IgG	C6-IgG	pepC10-IgM	VlsE1-IgM	VIDAS EIA
**Beta-coefficient**	0.045	0.284	0.037	0.118	0.402
**Standard Error**	0.015	0.044	0.009	0.025	0.195

^**a**^ Derived using normalized data from Bacon et al. [24].

Subsequent analyses were directed to empirically compare the sensitivity and specificity of diagnostic scores to binary models and the STT. Table 2 lists the performance of all binary models we evaluated, both as single-tier tests and as the second tier of a 2-tiered approach (i.e., MTT format); this list includes all possible combinations of kinetic-EIAs using “OR” logic. Of the 5 EIAs available for MTT approaches, the VIDAS EIA was the best performing first-tier assay. We explored using the C6-IgG antibody kinetic-EIA as the first-tier of a MTT approach, but this choice led to a 12% loss of sensitivity compared to VIDAS EIA-based MTT models (5) and (8) from Table 2; the latter 2 models were the most competitive assays that did not include the C6-IgG EIA in the second tier. Based on the above findings, all our MTT models utilized only a first-tier VIDAS EIA.

**Table 2:** Sensitivity and specificity of binary models constructed using Boolean “OR” logic^a^.

Binary antibody model (model number)^b^	Sensitivity	Specificity	Modified 2-tier sensitivity^c^	Modified 2-tier specificity^c^
**2-antibody models**				
**(1) VM, PM**	0.575	0.979	0.532	0.996
**(2) CG, VM**	0.725	0.979	0.681	0.995
**(3) VG, VM**	0.725	0.979	0.698	0.998
**(4) VG, CG**	0.750	0.979	0.718	0.993
**(5) VG, PM**	0.757	0.979	0.729	0.995
**(6) CG, PM**	0.779	0.979	0.743	0.991
**3-antibody models**				
**(7) VG, CG, VM**	0.782	0.968	0.732	0.993
**(8) VG, VM, PM**	0.804	0.968	0.754	0.995
**(9) CG, VM, PM**	0.811	0.968	0.757	0.991
**(10) VG, CG, PM**	0.814	0.968	0.771	0.989
**4-antibody models**				
**(11) VG, CG, VM, PM**	0.839	0.957	0.779	0.989
**(12) 2 or more positive: VG, CG, VM, PM** ^d^	0.693	1.00	N.A.	N.A.
**5-antibody model**				
**(13) 2 or more positive: VIDAS, VG, CG, VM, PM** ^e^	0.793	0.989	N.A.	N.A.

VG, VlsE1-IgG antibody; CG, C6-IgG antibody; PM, pepC10-IgM antibody; VM, VlsE1-IgM antibody; VIDAS, IgG and IgM antibodies to *Borrelia burgdorferi* by VIDAS EIA; N.A., not applicable

^**a**^ Database from Bacon et al. [24].

^b^ Binary models (1) through (11) were considered positive if any component antibody exceeded its individual cutoff.

^c^ Only samples positive or equivocal by VIDAS EIA were analyzed.

^d^ At least two antibodies must exceed their individual cutoffs to consider the overall test positive. A MTT model using these antibodies was unnecessary because model (12) specificity was already 100%.

^e^ At least two antibodies must exceed their individual cutoffs to consider the overall test positive. A MTT model was not possible because all available antibody assays were already utilized in the proposed format.

Specific binary models were selected as competitors to diagnostic scores and the STT based on their performance both as single-tier models and as MTT models. The most promising 2-antibody binary models identified by Bacon et al. [24] utilized pepC10-IgM antibody plus either VlsE1-IgG or C6-IgG antibodies [models (5) and (6), respectively, from Table 2]; when used in a MTT format, they were at least 99% specific. The most sensitive 3-antibody binary model utilized VlsE1-IgG, C6-IgG, and pepC10-IgM [model (10) from Table 2], but it failed to meet the 99% specificity cutoff in a MTT format. The most sensitive 3-antibody binary models that also demonstrated specificity ≥ 99% in a MTT format employed VlsE1-IgM, pepC10-IgM, and either VlsE1-IgG or C6-IgG antibodies [models (8) and (9), respectively, from Table 2]; these two models demonstrated equivalent overall performance and are discussed in detail below. The MTT version of model (8) is the most similar to the current FDA-cleared MTT assay [42]. The 4-antibody MTT assay [model (11) in Table 2], and the 5-antibody assay [model (13) from Table 2] both performed reasonably well, but also failed to meet the 99% specificity cutoff. Although models (10), (11), and (13) from Table 2 were competitive with diagnostic scores, all required additional tests or tiers to achieve slightly worse sensitivity and specificity than the 3-antibody diagnostic score.

Table 3 compares the performance of individual assays, single-tier binary models and diagnostic scores. Logistic-likelihood regression analysis was used to generate diagnostic scores for 2-antibody combinations and were then compared to their binary model counterparts. The diagnostic score employing VlsE1-IgG, C6-IgG, and pepC10-IgM antibodies with one interaction term was compared to its single-tier binary model counterpart [model (10) from Table 2], the STT, and other selected binary models. The 3-antibody diagnostic score using cutoff 1 was 12.1% more sensitive than the STT (95% CI: 7.1% to 17.2%), but equally specific (99.1%). Although the 2- and 3-antibody binary models listed in Table 3 were also significantly more sensitive than the STT, they were 1.3% to 2.3% less specific than both their corresponding diagnostic scores and the STT. To properly compare model specificities, their performance should be compared at equivalent sensitivity. The performance of the 3-antibody diagnostic score was therefore evaluated at 3 different cutoffs to generate sensitivity comparable to the 2- and 3-antibody binary models listed in Table 3; the specificity of the diagnostic score at each cutoff value was then statistically compared to that of its corresponding binary model (see Table 4). Depending on the cutoff used, the 3-antibody diagnostic score was 1.8% to 2.3% more specific than the binary models listed in Tables 3 and 4; unlike the above binary models, the 3-antibody diagnostic score was able to exceed STT sensitivity without any loss of specificity.

**Table 3:** Sensitivity and specificity of antibody response to recombinant and peptide antigens in 280 patients with Lyme disease and 559 controls^a^.

Antibody assays	Sensitivity (%)	Specificity (%)
Individual antibody assays		
C6-IgG (CG)	66.4	99.1
VlsE1-IgG (VG)	65.7	99.1
pepC10-IgM (PM)	38.2	99.1
VlsE1-IgM (VM)	36.4	99.1
VIDAS EIA	83.9	90.9
Combination antibody assays ^b^ ^,^ ^c^		
CG, PM (binary model)^d^	77.8	97.8
CG, PM (diagnostic score)	76.4	99.1
VG, PM (binary model)^e^	75.7	97.8
VG, PM (diagnostic score)	71.8	99.1
VG, CG, PM (binary model)	81.4	96.8
VG, CG, PM (diagnostic score)^f^		
Cutoff 1	79.6	99.1
Cutoff 2	77.8	99.6
Cutoff 3	75.7	99.6
**Standard 2-tiered method (STT)** ^g^	**67.5**	**99.1**

^**a**^ Database from Bacon et al. [24].

^**b**^ Binary models were considered positive if any component assay exceeded its individual cutoff.

^c^ The 2- and 3-antibody diagnostic scores consisted of weighted linear combinations of their corresponding antibody test results; only the 3-antibody diagnostic score included an interaction term.

^**d**^ The binary model using C6-IgG and pepC10-IgM antibodies was 10.4% more sensitive than the STT (95% CI: 5.1% to 15.6%).

^**e**^ The binary model using VlsE1-IgG and pepC10-IgM antibodies was 8.2% more sensitive than the STT (95% CI: 3.0% to 13.4%).

^**f**^ The 3-antibody diagnostic score using cutoff 1 was 12.1% more sensitive than the STT (95% CI: 7.1% to 17.2%) and equally specific.

^g^ Samples positive or equivocal by VIDAS EIA were confirmed by IgG and IgM immunoblots interpreted using CDC guidelines [3].

**Table 4:** Comparative specificity of multi-antibody assays at equivalent sensitivity.

Model comparisons^a^^,^^b^	Sensitivity	Sensitivity difference	Specificity	Specificity difference	95% CI of specificity difference
**3-Ab DS (1)****vs**. **VG, CG, PM (binary)**	79.6% vs. 81.4%	-1.8% (NS)^c^	99.1% vs. 96.8%	2.3% (S)	1.1% to 4.0%
**3-Ab DS (2)****vs**. **CG, PM (binary****)**	Both 77.8%	None	99.6% vs. 97.8%	1.8% (S)	0.8% to 3.3%
**3-Ab DS (3)****vs**. **VG, PM (binary)**	Both 75.7%	None	99.6% vs. 97.8%	1.8% (S)	0.5% to 3.4%

vs, versus; 3-Ab DS (1), (2), or (3), 3-antibody diagnostic score using cutoffs (1), (2), or (3), respectively, as reported in Table 3; (binary), binary antibody model utilizing the listed antibodies; CI, confidence interval; VG, VlsE1-IgG antibody; CG, C6-IgG antibody; PM, pepC10-IgM antibody; NS, not statistically significant; S, statistically significant.

^**a**^ The 3-antibody diagnostic score (3-Ab DS) consists of a weighted linear combination of VlsE-IgG, C6-IgG, and pepC10-IgM antibody levels with one statistical interaction term; its performance is reported at 3 different cutoff levels.

^**b**^ Binary models were considered positive if any component assay exceeded its individual cutoff.

^**c**^ 95% CI of difference in sensitivity: - 0.4% to 4.1%.

Table 5 provides disease stage-specific comparisons of STT performance versus select binary models, and the 3-antibody diagnostic score. The 3-antibody diagnostic score using cutoff 1 was 22.5% more sensitive than the STT for early-acute-phase disease (95% CI: 11.8% to 32.2%), 16.0% more sensitive for early-convalescent-phase disease (95% CI:7.2% to 24.7%) and was equally sensitive for disseminated infection (92.6% versus 93.6%, respectively); assay specificity was identical (99.1%). The sensitivity of the 3-antibody DS using cutoff 1 was statistically equivalent to the 2- and 3-antibody binary models in Table 5 for both early LD and disseminated infection, but was 1.3% to 2.3% more specific. Unless a new assay is at least as specific as the STT, confirmatory testing of positive or equivocal first-tier results is recommended [21, 30, 63]. Any specificity loss relative to the STT, such as that observed using the binary antibody models described in Tables 3–5, would likely have clinical import given the extensive overuse of serology in the US [17, 48, 65]; for every 1% loss of specificity, at least 30,000 additional false-positive results might be generated [48]. In order to improve the specificity of binary models, we explored modified 2-tiered approaches that demonstrated specificity greater than or equal to the STT method (See Fig 1, Tables 2 and 6). The best performing MTT binary models utilized VlsE1-IgM, pepC10-IgM, and either VlsE1-IgG or C6-IgG antibodies for confirmation of positive or equivocal VIDAS EIA results [Table 2, models (8) and (9), respectively, and Table 6]. Model (8), the MTT binary model most similar to the current FDA-cleared MTT assay, demonstrated significantly better overall sensitivity than the STT and the MTT using C6-IgG antibody alone for diagnosis of early Lyme disease. For early-acute-phase disease the MTT using VlsE1-IgG, VlsE1-IgM, and pepC10-IgM antibodies was 13.8% more sensitive than the STT (95% CI: 4.0% to 22.9%) and 12.5% more sensitive that the MTT using C6-IgG alone (95% CI: 3.7% to 20.9%). For early-convalescent-phase disease, the MTT using VlsE1-IgG, VlsE1-IgM, and pepC10-IgM antibodies was 9.4% more sensitive than the STT (95% CI: 0.9% to 17.9%) and 8.5% more sensitive than the MTT using C6-IgG alone (95% CI: 0.6% to 16.4%). The sensitivity of all 2- and 3-antibody MTT assays in Table 6 for disseminated infection was comparable to the STT. We detected no advantage to using the 3-antibody diagnostic score in a MTT format. The specificities of the 3-antibody diagnostic score using cutoff 1 and its MTT version were statistically equivalent (99.1% versus 99.6%, respectively; 95% CI of difference: 0.2% to -1.6%) and the sensitivity of the 3-antibody DS using cutoff 1 appeared slightly higher than its MTT version (79.6% versus 76.8%, respectively, Tables 5 and 6). Subsequent analyzes compared the sensitivity of the single-tier 3-antibody diagnostic score from Table 5 to that of select MTT binary models (Table 7).

**Table 5:** STT performance versus C6-IgG, select binary models, and the 3-antibody diagnostic score^a^^,^^b^.

Disease Stage	No. of sera	STT No.+ (%)	CG No.+ (%)	CG, PM (binary) No.+ (%)	VG, VM, PM (binary) No.+ (%)	CG, VM, PM (binary) No.+ (%)	3-Ab DS No.+ (%)
**Total**	280	189 (68)	186 (66)	218 (78)	225 (80)	227 (81)	223 (80)
**Early LD**	186	101 (54)	110 (59)	135 (73)	136 (73)	142 (76)	136 (73)
**EA**	80	30 (38)	36 (45)	50 (63)	47 (59)	50 (63)	48 (60)
**EC**	106	71 (67)	74 (70)	85 (80)	89 (84)	92 (87)	88 (83)
**Disseminated LD**	94	88 (94)	76 (81)	83 (88)	89 (95)	85 (90)	87 (93)
**Neuro**	37	33 (89)	24 (65)	31 (84)	35 (95)	32 (86)	33 (89)
**AT**	57	55 (96)	52 (91)	52 (91)	54 (95)	53 (93)	54 (95)
**Controls**	559	5 (1)	6 (1)	12 (2)	18 (3)	18 (3)	5 (1)
**Anti-cardiolipin**	15	0	0	0	0	0	0
**ANA**	116	0	2	6	6	7	1
**Endemic**	14	0	0	0	6	0	1
**Non-endemic**	243	0	0	0	1	5	0
**Lepto**	10	1	2	2	1	2	1
**MS**	10	0	0	0	0	0	0
**RPR +**	14	0	0	0	1	0	0
**RA**	94	1	0	1	2	1	0
**RF +**	15	0	0	0	0	0	0
**Syphilis**	14	1	1	1	0	1	1
**TBRF**	14	2	1	2	1	2	1

No.+, number of sera positive by a given model; (%), percent positive; Total, all samples tested; STT, standard 2-tiered method; CG, C6-IgG antibody; VG, VlsE1-IgG antibody; VM, VlsE1-IgM antibody; PM, pepC10-IgM antibody; (binary), binary model utilizing the listed antibodies; 3-Ab DS, 3-antibody diagnostic score; Early LD, both early-acute-phase and early-convalescent phase sera from patients with erythema migrans; EA, early-acute-phase sera; EC, early-convalescent-phase sera; Disseminated LD, neuroborreliosis and Lyme arthritis; Neuro, neuroborreliosis; AT, Lyme arthritis; Anti-cardiolipin, anti-cardiolipin antibodies; ANA, anti-nuclear antibodies; Lepto, leptospirosis; MS, multiple sclerosis; RPR +, rapid plasma regain-positive; RA, rheumatoid arthritis; RF+, rheumatoid factor positive; TBRF, tick-borne relapsing fever. See Methods for additional model descriptions.

^**a**^ The 3-antibody diagnostic score (3-Ab DS) uses cutoff 1 (Table 3). 3-Ab DS specificity was 1.3% better than the binary model using C6-IgG and pepC10-IgM antibodies (95% CI: 0.2% to 2.6%) and 2.3% better than the 3-antibody binary models using VlsE-IgM, pepC10-IgM, and either VlsE1-IgG or C6-IgG antibodies (95% CI: 0.6% to 4.2%, and 1.0% to 3.7%, respectively).

^**b**^ Binary models were considered positive if any component assay exceeded its individual cutoff value.

**Table 6:** STT performance versus modified 2-tiered approaches using C6-IgG, select binary models, and the 3-antibody diagnostic score as second-tier assays^a^^,^^b^.

Disease Stage	Total Sera (No. VIDAS EIA + or equivocal)	STT No.+ (%)	CG (MTT) No. + (%)	CG, PM (MTT) No.+ (%)	VG, VM, PM (MTT) No.+ (%)	CG, VM, PM (MTT) No.+ (%)	3-Ab DS (MTT) No.+ (%)
**Total**	280 (235)	189 (68)	179 (64)	208 (74)	211 (75)	212 (76)	215 (77)
**Early LD**	186 (142)	101 (54)	103 (55)	125 (67)	122 (66)	127 (68)	128 (69)
**EA**	80 (47)	30 (38)	31 (39)	42 (53)	41 (51)	42 (53)	42 (53)
**EC**	106 (95)	71 (67)	72 (68)	83 (78)	81 (76)	85 (80)	86 (81)
**Disseminated LD**	94 (93)	88 (94)	76 (81)	83 (88)	89 (95)	85 (90)	87 (93)
**Neuro**	37 (37)	33 (89)	24 (68)	31 (84)	35 (95)	32 (86)	33 (89)
**AT**	57 (56)	55 (96)	52 (91)	52 (91)	54 (95)	53 (93)	54 (95)
**Controls**	559 (51)	5 (1)	3 (0.5)	5 (1)	3 (0.5)	5 (1)	2 (0.4)
**Anti-cardiolipin**	15 (0)	0	0	0	0	0	0
**ANA**	116 (7)	0	0	0	0	0	0
**Endemic**	14 (2)	0	0	0	1	0	0
**Non-endemic**	243 (9)	0	0	0	0	0	0
**Lepto**	10 (3)	1	1	1	0	1	0
**MS**	10 (0)	0	0	0	0	0	0
**RPR +**	14 (5)	0	0	0	0	0	0
**RA**	94 (4)	1	0	1	1	1	0
**RF +**	15 (2)	0	0	0	0	0	0
**Syphilis**	14 (11)	1	1	1	0	1	1
**TBRF**	14 (8)	2	1	2	1	2	1

(MTT), modified 2-tiered approach using the listed binary antibodies or diagnostic score; STT, standard 2-tiered method; (No. VIDAS EIA + or equivocal), number of samples where the VIDAS EIA is positive or equivocal; 3-Ab DS, 3-antibody diagnostic score. See Table 5 for additional abbreviations.

^**a**^ Except for the STT, all models represent MTT approaches wherein samples positive or equivocal by a first-tier VIDAS EIA were confirmed by their respective second-tier assays. MTT models using binary antibodies were considered positive if any component assay exceeded its individual cutoff and the VIDAS EIA was either positive or equivocal. The MTT using the 3-antibody DS employed cutoff 1.

^**b**^ The MTT using the 3-Ab DS was 9.3% more sensitive overall than the STT and 12.9% more sensitive overall than the MTT using C6-IgG alone (*P*≤ 0.01 for each comparison by the Wilson method); significant differences in sensitivity for early-acute-phase and early convalescent-phase disease were also noted between the MTT using the 3-Ab DS and either the STT or the MTT using C6-IgG antibody alone (*P*≤ 0.01 for each comparison by the Wilson method). There were no statistical differences in specificity among the above models.

**Table 7:** Sensitivity of the 3-antibody diagnostic score versus MTT binary models at equivalent specificity^a^.

Model	No.	Sensitivity	Difference in sensitivity	95% CI of difference	Specificity	Difference in specificity
**3-Ab DS vs**. **CG (MTT)**	280	79.6% vs. 63.9%	15.7% (S)	11.3% to 20.1%	99.1% vs. 99.5%	-0.4% (NS)^b^
**3-Ab DS vs**. **CG (MTT) for EA**	80	60.0% vs. 38.8%	21.3% (S)	12.1% to 29.6%	99.1% vs. 99.5%	-0.4% (NS)
**3-Ab DS vs**. **CG (MTT) for EC**	106	83.0% vs. 67.9%	15.1% (S)	8.3% to 22.2%	99.1% vs. 99.5%	-0.4% (NS)
						
**3-Ab DS vs**. **VG, VM, PM (MTT)**	280	79.6% vs. 75.4%	4.3% (S)	1.2% to 7.5%	99.1% vs. 99.5%	-0.4% (NS)^c^
**3-Ab DS vs**. **VG, VM, PM (MTT) for EA**	80	60.0% vs 51.3%	8.7% (S)	1.7% to 15.5%	99.1% vs. 99.5%	-0.4% (NS)
**3-Ab DS vs**. **VG, VM, PM (MTT) for EC**	106	83.0% vs. 76.4%	6.6% (S)	1.1% to 12.5%	99.1% vs. 99.5%	-0.4% (NS)

vs, versus; (MTT), modified 2-tiered binary model utilizing the listed antibodies; EA, early-acute-phase sera; EC, early-convalescent-phase sera. See Table 5 for additional abbreviations and Table 6 for descriptions of MTT binary models.

^**a**^ The 3-antibody diagnostic score (3-Ab DS) using cutoff 1 was employed as a single-tier approach.

^**b**^ 95% CI: -1.4% to 0.5%.

^c^ 95% CI: -1.6% to 0.8%.

The single-tier 3-antibody DS detected significantly more cases of early-acute-phase and early-convalescent-phase disease than either the MTT using C6-IgG antibody alone or the MTT using VlsE1-IgG, VlsE1-IgM and pepC10-IgM antibodies. For diagnosis of disseminated infection the sensitivity of the 3-antibody DS was equivalent to the selected 2- and 3-antibody MTT binary models in Table 6, but superior to the MTT using C6-IgG antibody alone (92.6% versus 80.9%; 95% CI of difference: 4.6% to 19.7%). This single-tier 3-antibody DS was also 7.5% more sensitive for early-acute-phase disease than MTT binary model (9) using C6-IgG, VlsE1-IgM, and pepC10-IgM antibodies (60.0% versus 52.5%, 95% CI of difference: 1.8% to 13.1%), but was equivalent for diagnosis of early-convalescent-phase disease (83.0% versus 80.2%) and disseminated infection (92.6% versus 90.4); test specificity was identical (99.1%).

In summary, the MTT model using C6-IgG antibody alone did not perform as well as 2- and 3-antibody MTT binary models using our dataset. A single-tier 3-antibody diagnostic score performed as well as or better than MTT binary models using the same antibody panel, particularly for early-acute-phase disease; specificity was ≥ 99%.

A limited evaluation of disease stage-specific models did not identify diagnostic weights that improved assay sensitivity relative to the model trained on the entire dataset (Table 8); the specificity of all models was identical (99%). It is possible that a larger study or broader antibody panel might have been helpful in identifying stage-specific models, but could not be explored using the existing database.

**Table 8:** Performance of STT, 3-Ab DS trained on entire dataset, and disease stage-specific 3-Ab DS.

Model	EA	(%)	EC	(%)	Neuro/AT	(%)	Total	(%)
STT								
Sensitivity	30/80	38%	71/106	67%	88/94	94%	189/280	68%
3-Ab DS (overall)^a^								
Sensitivity	49/80	61%	87/106	82%	87/94	93%	223/280	80%
Stage-specific 3-Ab DS^b^								
Sensitivity	51/80	64%	87/106	82%	87/94	93%	225/280	80%

3-Ab DS, 3-antibody diagnostic score; Neuro/AT, combined neurological LD and Lyme arthritis; EA, early-acute-phase disease; EC, early-convalescent-phase disease

^**a**^ Beta-coefficients for VlsE-IgG, C6-IgG, and pepC10-IgM antibodies, as well as one interaction term, were derived using the entire dataset; specificity was 99% using cutoff 1. 3-Ab DS performance is reported for each disease stage. Statistical comparisons of the 3-Ab DS using cutoff 1 versus the STT are reported in Table 3 and the text.

^**b**^ Beta-coefficients for VlsE-IgG, C6-IgG, and pepC10-IgM antibodies, as well as one interaction term, were determined separately for each disease stage by logistic-likelihood regression analysis (see Methods); cutoffs were set for each disease stage-specific formula to generate 99% specificity among controls. Total performance represents the sum of the performances of each stage-specific diagnostic score.

We also analyzed our dataset using the partial ROC regression technique we described in a prior publication [48]; utilizing these same 3 antibodies and one interaction term we modified the beta-coefficients to maximize the portion of the ROC curve between 80% and 100% specificity. We then compared the partial AUC between 80% and 100% specificity generated through our logistic-likelihood regression model to the partial ROC regression method and found no significant difference (0.1794 versus 0.1797, respectively). We cannot, however, exclude a possible advantage to applying partial ROC regression techniques to other patient populations or diagnostic antibody panels.

## Discussion

Using modern bioinformatic techniques, we reanalyzed the serologic database from a previously published study by Bacon et al. [24] that utilized recombinant VlsE1, C6 peptide, and pepC10 antigens, as well as whole-cell lysate, for Lyme disease serodiagnosis; this study represents the largest publicly-available database of LD serodiagnostics in the US, and its size permitted a detailed examination of the immune response at different disease stages. Using logistic-likelihood regression modeling and ROC techniques we determined that VlsE1-IgG, C6-IgG, and pepC10-IgM antibodies, as measured by a kinetic-EIA, provided complementary diagnostic information. A diagnostic score was generated for each patient’s sample using a weighted linear combination of antibody levels to these 3 antigens. Compared to the STT, a single-tier 3-antibody diagnostic score (DS) was 22.5% more sensitive for early-acute-phase LD, 16.0% more sensitive for early-convalescent-phase disease, comparable for diagnosis of disseminated infection, and equally specific.

Given the overuse of serology in the US, any specificity loss greater than 1% relative to the STT would likely be clinically significant [48, 63]. The specificity loss associated with all single-tier multi-antibody binary models we evaluated was unacceptably high, so we explored MTT approaches that met or exceeded STT specificity. The MTT binary models utilizing VlsE1-IgM, pepC10-IgM, and either VlsE1-IgG or C6-IgG antibodies demonstrated significantly better overall sensitivity than the STT while maintaining equivalent specificity; these results were particularly applicable to patients with early-acute-phase LD. The single-tier 3-antibody DS, however, demonstrated better sensitivity than the latter two MTT models for early-acute-phase LD. Alternative MTT binary models with specificity near but not equal to STT specificity could approximate the performance of the 3-antibody DS, but required either more tests or more tiers to do so; none demonstrated better overall sensitivity than the diagnostic score.

Our observations demonstrated the ability of diagnostic scores to improve either the sensitivity or specificity of multi-antibody assays; these findings are consistent with bioinformatic theory [49] and with prior studies using diagnostic scores to interpret multi-antibody assays [34, 48]. Although the present study evaluated multiple kinetic-EIA antibody assays performed separately, this multi-antibody approach could be adapted to a multiplex platform using equally sensitive technology (e.g., microsphere and biochip assays); these multiplex platforms hold the potential for simpler and faster Lyme disease serodiagnosis [48, 54, 66]. While multiplex assay standardization presents technical challenges [67], a diagnostic score can still be utilized effectively based on the same mathematical principles [48].

Bacon et al. [24] observed that the sensitivity of their VlsE1-IgG kinetic-EIA was greater than the C6-IgG kinetic-EIA in their 37 patients with neuroborreliosis (89% versus 65%, respectively). Other investigators have reported variable results concerning the relative sensitivity of IgG antibodies to VlsE and C6 for neuroborreliosis. Liang et al. [25] reported 95% sensitivity using a C6-IgG EIA in their 20 patients with early neuroborreliosis. Both Ledue et al. [68] and Pegalajar-Jurado et al. [29] observed that the sensitivity of the Oxford Immunotec C6 IgG/IgM EIA appeared equivalent to the Liaison^®^ VlsE-IgG/IgM chemiluminescent assay for early neurological disease. A recently developed FTIS biochip immunoassay by Huang et al. [66] demonstrated better sensitivity using VlsE-IgG than C6-IgG in 56 patients with neuroborreliosis (75% versus 54%, respectively). Although the Bacon, Liang, and Huang studies used the same C6 sequence from *Borrelia garinii*, different assay techniques and patient populations may have contributed to differences in assay sensitivity for neuroborreliosis. Assay sensitivity alone may not be sufficient to assess the diagnostic potential for IgG antibodies to these two antigens. A 2007 study by Embers et al. [27] demonstrated than fewer than half of LD sera demonstrating IgG antibodies to both recombinant VlsE and C6 could have their IgG antibody binding to recombinant VlsE competitively blocked by adding C6 peptide to the samples; this observation suggests the presence of a VlsE epitope other than C6 that binds IgG antibody. Combined with the current study, the above data suggest that IgG antibodies to VlsE and C6 may be diagnostically complementary, at least in some patient populations.

MTT approaches that utilize kinetic-EIAs to detect either C6-IgG antibody or a combination of VlsE1-IgG, VlsE1-IgM and pepC10-IgM antibodies are similar to but technically different from MTTs that have used either the Oxford Immunotec C6 EIA for IgG/IgM antibodies or the Zeus EIA for IgG and IgM antibodies to VlsE1 and pepC10. Although both commercial assays detect polyvalent antibodies to their respective antigens, prior investigations suggest that assays for IgM antibody to C6 and IgG antibody to pepC10 using standard EIAs add little toward diagnosis [26, 27, 69]. Consistent with prior studies, the C6-IgG kinetic-EIA we evaluated detected more cases of early-acute-phase disease than the STT [21]; its performance was equivalent, however, to the STT when used in a MTT format. In contrast, other investigators have noted better sensitivity for early LD when using MTT approaches that employ a second-tier EIA for IgG/IgM antibodies to C6 [5]. Because of differences in assay techniques and patient populations, only head-to-head comparisons of kinetic-EIAs to commercial products using the same population group can properly evaluate their relative performance. The primary purpose of our study was not to compare kinetic-EIA assays to commercial assays; utilizing the assays available to us, we explored different single-tier and MTT approaches to find to the best performing alternatives to the STT. Our findings suggest that a single-tier 3-antibody kinetic-EIA assay, interpreted using a diagnostic score, may provide a promising alternative to both STT and MTT approaches for diagnosis of early LD.

Our ability to generalize our findings is limited by the retrospective study design, diagnostic criteria, study location, distribution of disease manifestations, duration of infection and antibiotic treatment prior to testing, and the age of the samples tested. Only 55% of early LD sera were from patients with culture-proven EM; therefore, some EM lesions might have been due to other causes, such as STARI. STARI is not known, however, to cause cross-reacting antibodies to *B*. *burgdorferi* [12] and is therefore unlikely to explain differences in assay performance. Twenty-four percent of our study population consisted of patients with late-stage neuroborreliosis or arthritis; in contrast, population-based studies suggest that fewer than 10% of patients now present with extracutaneous disease [8, 70]. Because the study definition of late-stage LD included positive STT serology, both patient selection bias and the case definition likely favored the STT approach. Forty-eight percent of early-acute-phase sera came from patients with multiple EM lesions, in contrast to more recent experience suggesting that only 21% of patients with EM demonstrate multiple lesions [71]; it is known that the STT is more often positive in patients with multiple EM [72, 73], potentially enhancing its performance relative to C6 antibody assays [73]. Despite the retrospective study design, 46% of pre-treatment, early-acute-phase sera were collected within 1 week of disease onset and 85% within 30 days; these early-acute-phase patients are more representative of cases seen in general practice than the other disease stages we studied. The 26 early-convalescent-phase sera contributed by the CDC (out of 106 total cases) were obtained a median of 55 days after starting antibiotics, possibly limiting antibody responses [7, 72]; although the latter patients may experience loss of antibody to VlsE1 in particular [68], the 3-antibody DS model still demonstrated improvement relative to the STT and the MTT using C6-IgG antibody alone in early-convalescent-phase sera. When performed in conjunction with early-acute-phase serology, early-convalescent-phase serology obtained within 2 to 3 weeks of beginning antibiotic treatment may still be important when the diagnosis is uncertain or to confirm infection in areas where LD is an emerging pathogen. No patients with “influenza-like” disease without EM were studied, so the performance of the 3-antibody DS in this group is unknown. More than 95% of LD sera in our database were collected in the Northeastern and mid-Atlantic US; although there may be genotypic differences in *B*. *burgdorferi* between regions [74], disease manifestations are not known to be region specific. Sera from patients with late-stage disease had been collected over a 20-year period before testing, potentially limiting serologic responses due to antibody degradation. Although the VIDAS Lyme IgG and IgM (LYT) EIA is no longer commercially available, its performance is equivalent to the newer VIDAS Lyme IgM II and IgG II EIAs that detect IgG and IgM antibodies to *B*. *burgdorferi* separately (bioMerieux Vitek system, Durham, NC) [75]. Two samples may have been misclassified by Bacon et al. [24] as STT-positive based on IgM blot criteria alone (see Methods above); reclassifying these patients as STT-negative did not affect our study conclusions.

Study patients with LD were not systematically screened for tick-borne co-infections; we now know that human granulocytic anaplasmosis (HGA) can lead to cross-reacting IgM antibodies to *B*. *burgdorferi* [76], and *Borrelia miyamotoi* infection can cause false-positive reactions to the C6 peptide and whole-cell EIAs [77]. A recent prospective study by Wormser et al. [78] suggested that fewer than 10% of patients with erythema migrans demonstrate concurrent tick-borne co-infections (including *B*. *miyamoto*i). A seroprevalence study conducted by Krause et al. [79] in the Northeastern US revealed that 9.8% of patients with acute Lyme disease demonstrated antibodies to *B*. *miyamotoi*; this same study noted that among 36 patients seropositive for *B*. *miyamotoi* but without recognized Lyme disease during the prior 2 years, 6 (17%) demonstrated antibodies to the C6 peptide and 4 (11%) were STT positive. Extrapolating from the above seroprevalence studies, it is possible that 3% of our early-acute-phase sera might have demonstrated false-positive serology to *B*. *burgdorferi* as a consequence of either past or concurrent *B*. *miyamotoi* infection; we therefore believe that is unlikely that cross-reactive antibodies due to *B*. *miyamotoi* explain the 23% improvement in sensitivity for early-acute-phase disease demonstrated by the 3-antibody DS relative to the STT or the 15% improvement in sensitivity relative to C6-IgG antibody in this population. Described only in the last decade in the upper Midwestern US, *Borrelia mayonii* infection is a rare cause of Lyme disease and can also induce antibodies to the C6 peptide [80–82]. Since the vast majority of LD samples in our database were collected in the Northeastern and mid-Atlantic US, and since all were collected prior to 2003, it is unlikely that *B*. *mayonii* affected our study results [82].

Our control population included only 14 patients from Lyme disease-endemic communities, a potential source of false-positive results related to previous LD. Most false-positive STT serology, however, is due to IgM immunoblotting [6, 48]. The paradigm we have proposed reports a total score, not results from individual components, so cross-reactions due specifically to IgM antibodies might not be recognized. The latter concern also applies to MTT assays that measure IgM antibodies to the C6 peptide or OspC-related epitopes (e.g., pepC10). Our control group did not include patients with diseases known to generate cross-reacting IgM antibodies to OspC epitopes, such as Epstein-Barr virus and HGA [7, 26, 76]. Most early disseminated infections generate IgM antibodies to *B*. *burgdorferi*, sometimes constituting the sole host immune response [19, 20]; therefore cross-reacting antibodies to OspC epitopes, including pepC10, may limit the diagnosis of early neurological and cardiac disease. It may not be safe, however, to require IgG immunoblot confirmation of first-tier assays for early neurological disease because of limited immunoblot sensitivity [20, 48]. Confirmation of positive 3-antibody DS results using an IgG-based antibody assay might be justified for diagnosis of late disseminated infection, such as Lyme arthritis and late neurological disease, since the latter conditions are more serious and often require IV antibiotics. We therefore evaluated an IgG-based diagnostic score that utilized only the VlsE1-IgG and C6-IgG antibody components for diagnosis of disseminated infection; after excluding the contributions of pepC10-IgM antibody and the interaction term in the diagnostic score, we classified samples whose diagnostic score still exceeded the original cutoff as positive. The specificity of an IgG-based DS was not significantly different from the DS using both antibody isotypes (99.6% versus 99.1%). Sensitivity of the IgG-based DS for both early and late disseminated disease was similar to the original 3-antibody DS. Only one patient with early convalescent neurological disease was missed by the IgG-based DS; the latter patient demonstrated a negative IgG immunoblot and negative IgG-based diagnostic score, but positive VIDAS EIA and IgM immunoblot. Also, one patient with convalescent Lyme arthritis would have been missed using an IgG-based DS; the latter patient demonstrated a positive VIDAS EIA and IgG immunoblot, but negative IgM immunoblot. Although IgG immunoblotting may be able to clarify discordant 3-antibody DS and IgG-based DS results for late disseminated infection, a blanket requirement for IgG immunoblot confirmation of all disseminated disease might underdiagnose early neuroborreliosis; since an IgG-based diagnostic score would require only an alternative interpretative standard rather than an entirely new assay, the latter approach may be a practical alternative to immunoblotting. Additional specificity studies are necessary before recommending confirmatory testing of the 3-antibody assay proposed in this study.

Although we were unable to develop disease stage-specific models, it is possible that a broader antibody panel or larger study might have demonstrated an advantage to this approach. The challenge to developing stage-specific diagnostic models is their complexity; laboratories typically receive no accompanying clinical information when samples arrive. Stage-specific reports would require application of different diagnostic scores for different disease stages; as discussed above, late-stage disease is more serious and might justify a specific report.

We did not consider cost-effectiveness when comparing diagnostic approaches, although diagnostic score cutoffs can be chosen to address this goal [49]. Nor did we address whether pre-test risk plays a role in optimizing either the choice of assays or their cutoffs. A formal decision analysis utilizing the current database is the subject of an ongoing study.

Standardization of multi-antibody assays provides both technical and biostatistical challenges [67, 83]. Although we employed a bootstrap cross-validation statistical technique to reduce overestimates of diagnostic score performance, a separate set of samples for assay validation may have provided a more accurate estimate of test performance. Our multi-antibody diagnostic score did not assign cutoffs to individual antibody tests, only to the overall score. Studies of multi-component (e.g., multiplex) assays have recognized the diagnostic value of individual test results that fall below the limit of quantitation (LOQ) [83]. Utilizing antibody levels below typical cutoffs, however, raises practical concerns about test precision, particularly if the coefficient of variation (CV) at the LOQ rises above 20%; no clear statistical guidance has emerged to address this issue, although substitution using a fraction of the LOQ has been suggested [83]. Intra-laboratory variation in multiplex results (or multi-antibody assays using the same format and equipment), may be related to changes in test environment or equipment that lead to correlated errors; the latter problem is more likely to impair test sensitivity than specificity [83]. Dessau et al. [45] suggested that the reproducibility of multi-antibody assays should focus on classification accuracy and that the variance of individual assay components may be less important. Because LD has multiple stages and disease manifestations, the goal of classification accuracy is more complex than a single binary answer. Ultimately stage-specific LD classifiers may be needed [84] and high variance of individual assays within a broader panel may limit test performance. The impact of utilizing test components that demonstrate CVs exceeding 20% needs greater study.

Newer approaches to LD diagnostics include transcriptomic and metabolomic assays [85, 86]; these assays detect dozens of separate biochemical and gene transcription responses to infection and use regression algorithms for test interpretation. Because transcriptomic and metabolomic host responses to infection begin almost immediately [86, 87], they hold promise for earlier diagnosis compared to antibody production and discrimination between active and inactive disease [87]. Identifying the most important diagnostic variables may be sensitive to the population used to standardize the assay, making it difficult to predict which variables will provide the broadest application for general use [88]. Better understanding of individual biochemical or gene transcription responses in disease pathogenesis may help narrow selection of diagnostic targets, aiding standardization [88]. The specificity of metabolomic and transcriptomic assays in control populations has been reported as between 90% and 95% [86, 87], raising questions about their potential impact in an age of overuse of LD assays [17, 23, 63]. Concerns about the precision of individual assay components and treatment of results at or below the limits of quantitation remain important questions to resolve before attempting to make clinical distinctions between active and inactive disease in specific patient populations.

The advantage of utilizing antibody assays, despite the lag in antibody production following disease onset, is the extent of information already known about *B*. *burgdorferi* disease pathogenesis and the host immune response [52, 89]. Intensive research over the last 20 years has identified broadly recognized in-vivo and in-vitro expressed antigens that generate antibody responses in most patients with Lyme disease (e.g., VlsE, C6, DbpA, BBK32, FlaB, and OspC) [52, 53, 89]. For patients living in endemic communities where physicians are confident in their diagnosis of EM, no serology is needed. Atypical cases of early LD, delayed diagnosis, early neurological disease without EM, and skin lesions consistent with EM in non-endemic areas may benefit from serology. Diagnosis of disseminated infection will likely remain dependent on serologic confirmation for the immediate future, although metabolomic and transcriptomic techniques may become useful additions to our diagnostic armamentarium. The current study demonstrates an advantage to using diagnostic scores for interpretation of multi-antibody assays for diagnosis of early LD; additional assay standardization through prospective studies is needed.

## Supporting information

S1 Data S1_Serologic data. (XLS)
